# Gastroblastoma: a case report and literature review

**DOI:** 10.3389/fonc.2024.1354021

**Published:** 2024-04-09

**Authors:** Jiayu Li, Gang Wang, Zhiwei Jiang

**Affiliations:** Department of General Surgery, Affiliated Hospital of Nanjing University of Chinese Medicine, Nanjing, Jiangsu, China

**Keywords:** gastroblastoma, gastric mass, epithelial and mesenchymal biphasic differentiation, MALAT1, GLI1

## Abstract

**Objective:**

Gastroblastoma is an extremely rare gastric tumor. Its pathogenesis remains unclear and there is a lack of specific clinical symptoms. The aim of this paper is to report a case of gastroblastoma and provide references for the diagnosis, treatment, and prognosis of this disease.

**Methods:**

The diagnosis and treatment of a 51-year-old female patient with gastroblastoma were retrospectively reported. Analyzing this case by combining the clinical data such as imaging and pathological results of patients with the relevant literature.

**Results:**

The patient’s chief complaint was the presence of melena persisted for over two weeks. Abdominal contrast-enhanced CT showed gastric antral nodules, and micro-probe endoscopic ultrasonography was considered as “gastric antral protruding lesions”. The initial diagnosis of “gastric stromal tumor” was made after admission, and surgical treatment was performed on September 23, 2021. Postoperative pathology showed that gastric mixed epithelial and stromal tumor, combined with immunohistochemical staining, was suggestive of gastroblastoma. No signs of tumor recurrence or metastasis were observed during the 2-year follow-up.

**Conclusion:**

Combined with the existing literature reports, the incidence of gastroblastoma is mainly higher in young men, and the predilection site is gastric antrum. The biological behavior of the tumor tends to be indolent, and the prognosis of most cases is favorable. However, due to the extremely small number of cases, this conclusion still needs a large number of cases and follow-up data to support. Postoperative pathological and immunohistochemical examination results are the only methods for definite diagnosis at present, and surgery is the first choice for treatment.

## Introduction

Gastroblastoma is a very rare low-grade malignant gastric tumor. Its histology is characterized by biphasic epithelial and mesenchymal differentiation. Miettinen et al. (1) reported the first case of gastroblastoma worldwide in 2009 and the first case of gastroblastoma in China was reported by Yangyang Ma et al. (2) in 2014. Due to the rarity of gastroblastoma and the variety of histological morphology, the diagnosis, treatment and prognosis of this disease are poorly documented.The clinical data of a patient with gastroblastoma were analyzed in this study, accompanied by a comprehensive review of the relevant literature. We investigated the clinicopathological features, diagnosis, differential diagnosis, treatment and prognosis of this gastroblastoma, in order to improve the comprehension among clinicians and pathologists while minimizing misdiagnosis.

## Case presentation

### Clinical features

**Figure 1:**
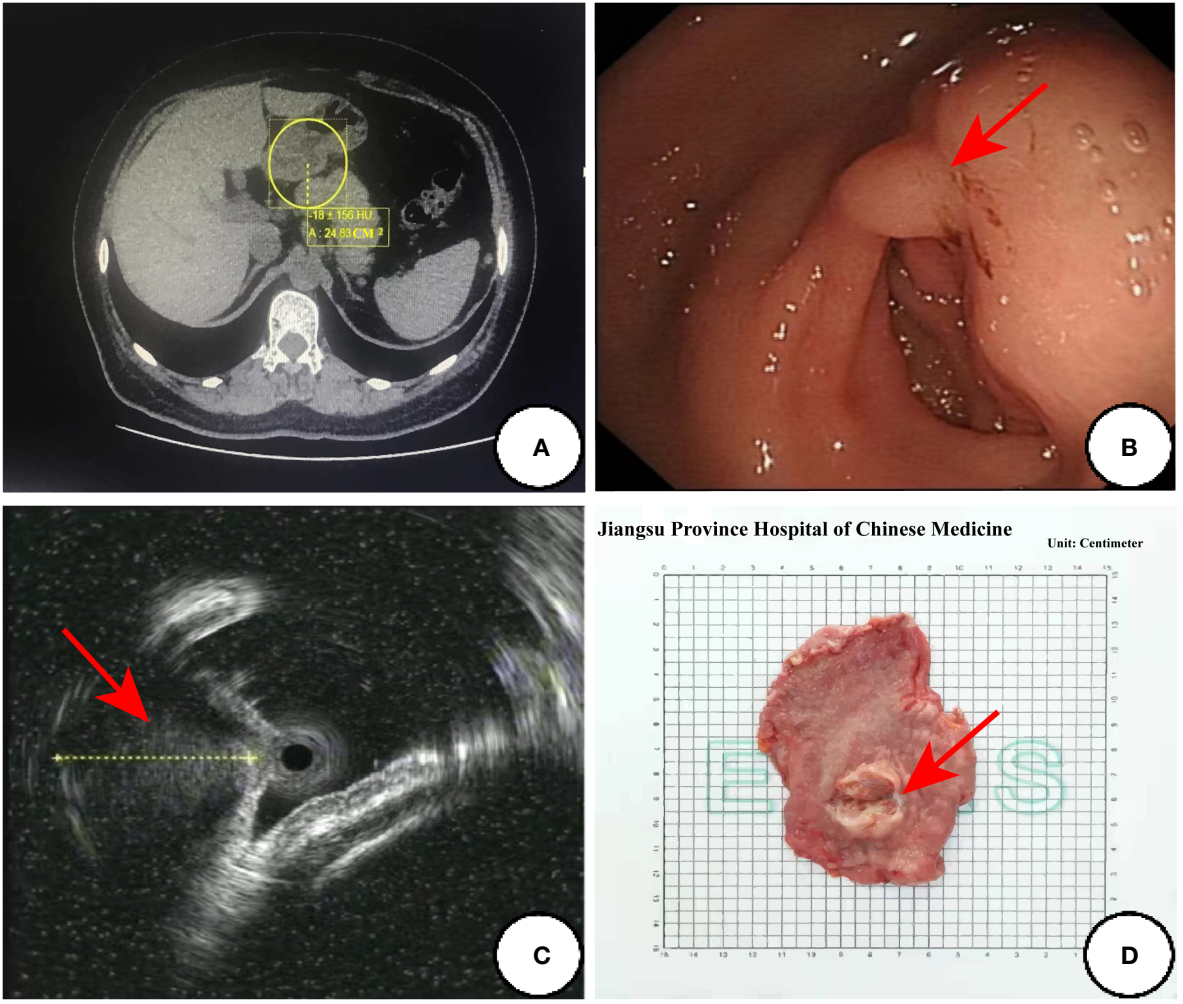
The preoperative examination results and surgical specimen. **(A)** Abdominal enhanced CT transverse axial view:a nodular object in the antrum was found in the yellow circle; **(B)** Endoscopic images of the ultrasonic gastroscope; **(C)** The ultrasound images of mini-probe endoscopic ultrasound: the red arrow indicates a lesion with a heterogeneous internal echo in antrum and the diameter of one section of the lesion was approximately 13.3mm; **(D)** Surgical specimen:the red arrow points to the tumor.

The 51-year-old female patient who was admitted to our hospital in September 2021,due to “the presence of melena over two weeks.” The contrast-enhanced abdominal CT from external hospital showed a nodule in the gastric antrum (**Figure 1A**). After admission, we conducted the requisite examinations for the patient. The endoscopic images obtained of endoscopic ultrasonography showed a deformed antrum with a 3.0 cm hemispherical uplift in the posterior wall of the stomach. In addition, the surrounding mucosal folds were found to be entangled under gastroscopy, and the biopsy forceps were hard to palpation (**Figure 1B**). The ultrasound images of mini-probe endoscopic ultrasound showed that the lesion was hypoechoic, the internal echo was not uniform, and the central part was hyperechoic, which originated from the muscularis propria layer and protrude into the cavity (**Figure 1C**). According to the patient’s symptoms and examination results, she was tentative diagnosed clinically with gastrointestinal stromal tumors(GIST), and the “Partial gastrectomy with gastrojejunostomy reconstruction” was performed on September 23, 2021 after excluding severe surgical contraindications.The surgical specimen was revealed to be a solid tumor of 2cm×3cm in size near the greater curvature of the antrum.The tumor grew within the wall of the stomach with a smooth surface and a tough texture (**Figure 1D**).

### Pathological examination

The resected gastric tissue was sent for examination and a hemispherical tumor was found in the posterior wall of the antrum which protruded into the cavity.The size of which was 2.8cm×1.8cm×1.5cm and its surface was covered with smooth gastric mucosa, and two small ulcers were focally found. On the cut surface, the solid tumor was gray-white and red in color, and the boundary is clear.

### Microscopy

**Figure 2:**
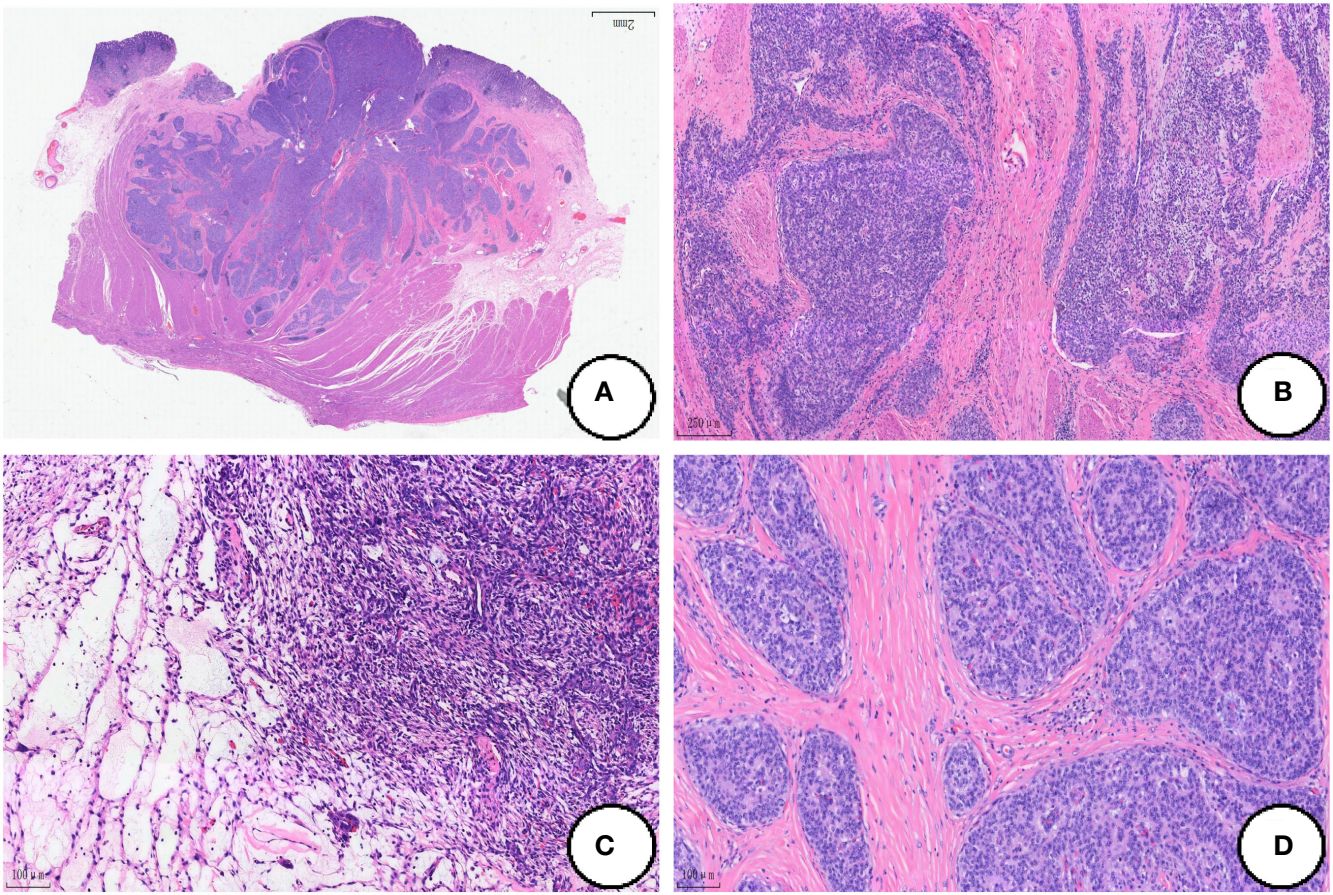
Histological findings of the tumor. **(A)** The tumor shows infiltrative growth in the mucosa, submucosa and muscularis propria; **(B)** The tumor is composed of epithelium and mesenchyma, which are promiscuously distributed but with clear boundaries; **(C)** The mesenchyme was composed of dense and sparse regions, and the oedematous changes can be dound in some regions; **(D)** The tumor cells are arranged in nets and cords in the the epithelioid regions. (Coloration HE, magnification×100 in **A**, **B**, magnification×200 in **C, D)**.

At low magnification, the tumor showed infiltrative growth in the mucosa, submucosa and muscularis propria (**Figure 2A**). The tumor was composed of epithelium and mesenchyma, which were promiscuously distributed but with clear boundaries (**Figure 2B**). The mesenchyme was composed of dense and sparse regions. The tumor cells were spindle-shaped and short spindle-shaped, with consistent morphology. Myxoid degeneration was observed in the stroma, and part of regions showed oedematous changes (**Figure 2C**).In the epithelioid regions, the tumor cells were arranged in nets and cords, and there was a tendency of glandular tube formation and mitotic Figures<5/50HPF (**Figure 2D**).

### Immunophenotype

**Figure 3:**
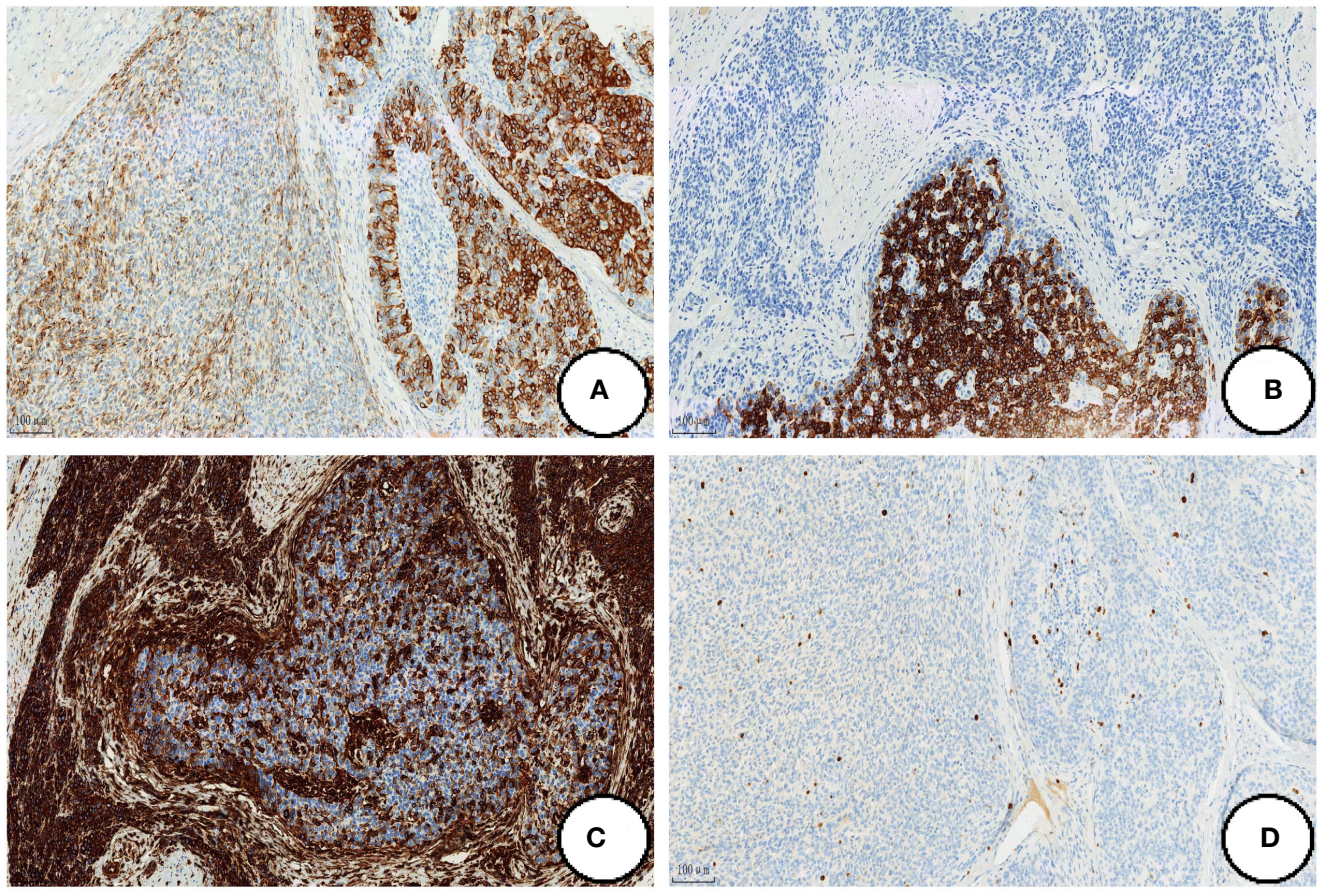
Immunohistochemical expression. The tumor cells strongly positive for Ckpan **(A)**,CK7 **(B)** and Vimentin **(C)**, and Ki-67 proliferation index was less than 5% **(D)**. (Coloration EnVision,magnification×200).

Epithelioid regional tumor cells expressed CKpan, CK7, EMA, CKL and CAM5.2, and CD56 was focally expressed. The tumor cells in the mesenchymal region were strongly positive for Vimentin and CD56 was also expressed in local (**Figures 3A-C**). In both epithelioid and mesenchymal regions, the tumor cells were negative for Desmin, DOG-1, CgA, SMA, Syn, CD-117, CK-20, CD10, CD34 and S-100, SDHB expression normally, and Ki-67 proliferation index was less than 5% (**Figure 3D**).

### Molecular pathology

**Figure 4:**
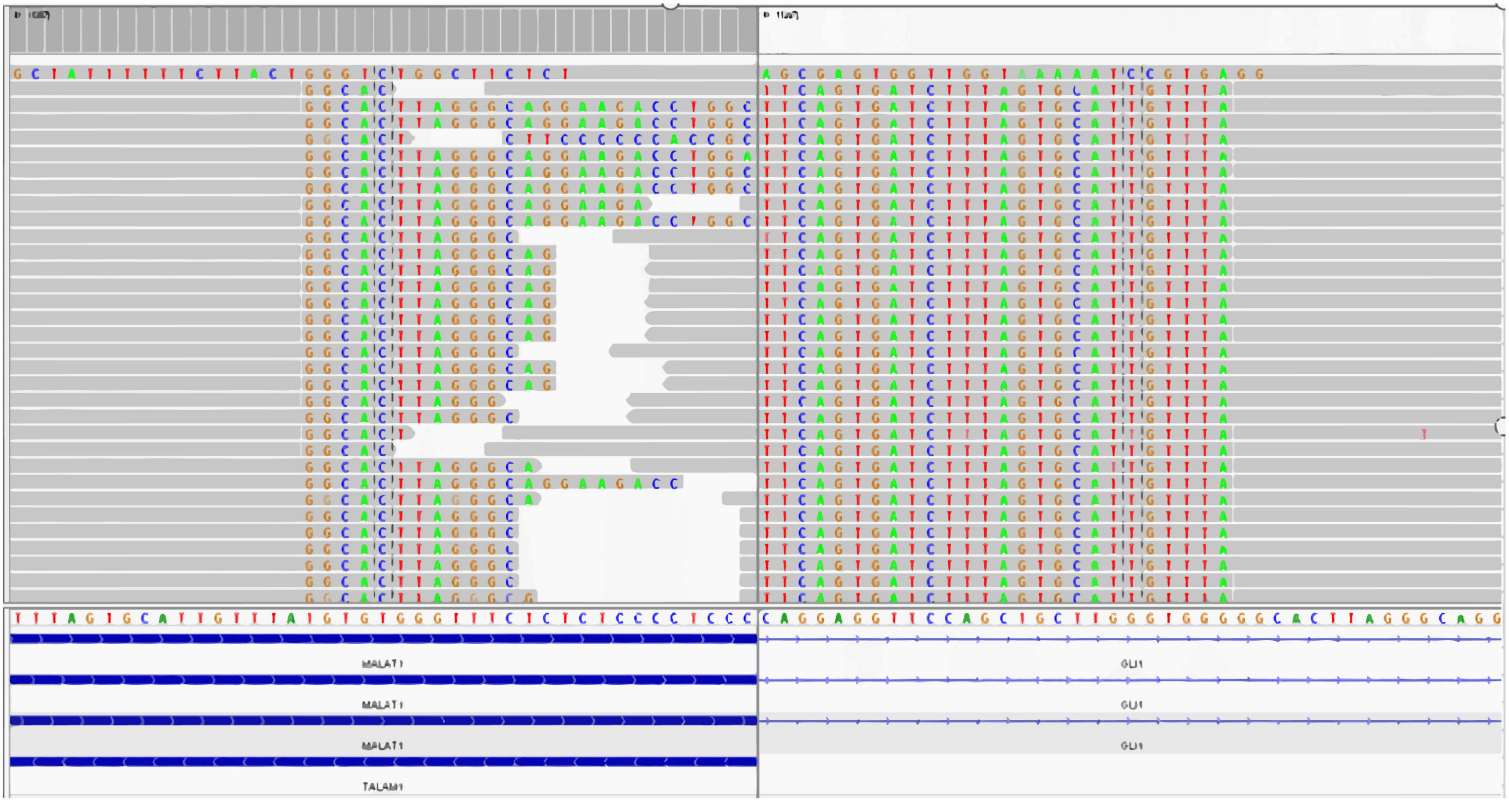
The fusion of MALAT1: exon1 and GLI1: exon6 is detected by the whole-transcriptome mRNA sequencing.

We performed whole-transcriptome mRNA sequencing of tumor tissue, which showed the fusion of MALAT1:exon1 and GLI1:exon6 (**Figure 4**).

### Pathologic diagnosis

The postoperative pathological results showed that the gastric epithelial-mesenchymal mixed tumor, combined with the immunohistochemical results, was inclined to gastroblastoma.

### Follow−up

This patient did not receive specific treatment after surgery, and no signs of recurrence or metastasis were observed during postoperative follow-up until September 23, 2023.

## Discussion

At the time of writing, 21 cases of gastroblastoma were retrieved in the medical literature. **Table 1** (1–18) summarizes the clinical features of these cases, including the present case. Among the 22 cases, there were 12 males and 10 females. The average age of onset was 36 ± 18.53 years old. In terms of gender, the incidence of male and female was similar, but in young patients who was under 30 years old, the incidence of male was higher (61.54%,8/13). Gastroblastoma tended to occur in the gastric antrum. Among the known cases, 11 cases occurred in the gastric antrum, and 4 cases occurred in the greater curvature of the gastric body. Gastroblastoma was characterized by gastrointestinal symptoms such as abdominal pain, abdominal mass and hematochezia, but lacked specific clinical manifestations. Gastroscopy and CT were the main methods for the diagnosis of gastroblastoma. In some cases, endoscopic ultrasonography was used to assist the diagnosis. Fernandes et al. (5) reported the imaging features of one patient in detail. CT of this case showed that the tumor was a lobulated cystic solid mass with a diameter of about 13cm, and the boundary of the tumor was relatively clear, with focal calcification. The contrast-enhanced CT showed moderate enhancement at the tumor margin and internal septum. Magnetic resonance(MR) showed cystic and solid lesions in the antrum of the stomach. The cysts varied in size, with a maximum diameter of about 8cm. The gross tumors of gastomoblastoma were mostly lobulated or nodular, mainly solid or cystic, and most of them were accompanied by hemorrhage and cystic lesions. The tumors showed infiltrative growth and could invade the whole layer of the gastric wall. In most reported cases, the cut surface could be greyish-white, greyish-red, greyish-yellow or hemorrhagic, and the maximum diameter ranged from 1.3cm to 15 cm. Histologically, the tumors were mainly composed of epithelioid and spindle cells. The epithelioid cells were well-circumscribed and arranged in sheets, nests or cord-tubular structures, while the spindle cells were short spindle-shaped or ovoid and arranged in whorls or loose networks. Both of them could be the main cellular components of tumors. The mitotic Figures of gastomoblastomas were mostly 0~5/50 HPF, and the mitotic Figures of some cases were active(30/50HPF) (1).

**Table 1:** Summary of published cases.

Case no.	Author (publication year)	Age (years)	Sex	Clinical features	Location	Size(cm)	Fusion gene	Treatment	Metastases/ relapse	Outcome	Follow up(month)
1	Miettnen et al. (2009) (1)	19	M	Nonspecific, abdominal pain	Greater curvature of the gastric body	5×4×2.5	ND	SG	NO	ANED	42
2	Miettnen et al. (2009) (1)	27	F	Nonspecific abdominal pain	Greater curvature of the gastric body	6×4×3.5	ND	PG	NO	ANED	60
3	Miettnen et al. (2009) (1)	30	M	Anemia, fatigue	Gastric antrum	15×12	ND	Antrectomy+ postoperative radiation	NO	ANED	168
4	Shin et al. (2010) (3)	9	M	Abdominal pain,periumbilical mass	Gastric antrum	9× 6.5	MALAT-GLI1 (Published in 2017 by Rondell P Graham)	Pargastric antrum+pylorus removed	NO	ANED	9
5	Wey et al. (2012) (4)	28	M	Constipation	Distal stomach	3.8×3.3×2.5	MALAT-GLI1 (Published in 2017 by Rondell P Graham)	Preoperative chemotherapy+PG	Liver, lymph node, retroperitoneal and bladder	ANED	3
6	Femandes et al.(2014) (5)	19	F	Abdominal pain	Gastric antrum	10.5	ND	Partial distal gastrectomy	NO	ANED	20
7	Yangyang Ma et al.(2014) (2)	12	M	Intermittent blood in stool, abdominal mass	Gastric antrum	4.5×2.5×2.5	ND	SG	NO	ANED	8
8	Toumi et al. (2017) (6)	29	F	epigastric pain	Greater curvature of the gastric body	7×4×4	ND	PG+ splenectomy	splenic, Lymph node/Local relapse	DIED	6
9	Graham et al. (2017) (7)	27	M	N/A	Gastric antrum	N/A	MALAT-GLI1	R	NO	ANED	12
10	Graham et al. (2017) (7)	56	F	N/A	Not specified	4	MALAT-GLI1	Needle Biopsy	Liver	N/A	N/A
11	Zhu Na et al. (2018) (8)	65	F	Intragastric mass	Greater curvature of the gastric body	1.3×1× 0.9	ND	EFTR	NO	ANED	3
12	Castri et al. (2019) (9)	79	M	Weight loss and dysphagia	Gastric antrum	9	MALAT-GLI1	PG	Local relapse	ANED	52
13	Centonze et al. (2019) (10)	43	F	Intestinal bleeding	Gastric antrum	5.3	ND	PG	NO	ANED	100
14	Long Weiguo et al. (2020) (11)	53	F	Abdominal pain	The junction of gastric antrum and gastric body	5× 4.2×4	ND	R	NO	ANED	14
15	Koo et al. (2021) (12)	17	M	Hematemesis and melena	Gastric cardia and fundus	6.3	EWSR1 -CTBP1	PG	NO	ANED	23
16	Cuimin Chen et al. (2022) (13)	58	M	N/A	Lesser curvature of the gastric body	2.43× 1.47	PTCH- GLI2	ESD	N/A	N/A	N/A
17	Chen Qi et al. (2022) (14)	43	M	Sour regurgitation and melena	Gastric antrum	4×2×2	ND	PG	NO	ANED	24
18	Li Guangliu et al. (2023) (15)	55	M	Gastric angle mass	Gastric angle	2× 1.8×1.5	ACTB- GLI1	ESD	NO	ANED	12
19	Sugimoto et al. (2023) (16)	28	F	Abdominal pain	Gastric antrum	7×7×6	MALAT-GLI1	PG	NO	ANED	8
20	Can Gong et al. (2023) (17)	19	F	Loss of appetite, and lost body weight	Gastric antrum	8.1×6.9×4.6	ND	PG	NO	ANED	19
21	McCammon et al. (2023) (18)	26	M	Abdominal pain	Gastric pylorus	6×5	MALAT–GLI1	R	NO	ANED	2
22	Our case	51	F	Melena	Gastric antrum	2.8×1.8×1.5	MALAT–GLI1	PG	NO	ANED	24

ANED, Alive no evidence of disease; EFTR, Endoscopic full-thickness resection; ESD, Endoscopic submucosal dissection; F, Female; M, Male; N/A, Not available; ND, Not done; PG, Partial gastrectomy; R, Resection; SG, Subtotal gastrectomy.

Immunohistochemically, the tumor cells in the epithelial region were strongly positive for CKpan, AE1/AE3, CAM5.2, CK7 and EMA and It also expressed CD56,CD10,CK(LMW) and CK18 to varying degrees. In the mesenchymal region, the tumor cells were strongly positive for Vimentin, and it also expressed CD56 and CD10 to varying drgrees. The tumor cells were usually negative for CD117, DOG1, CD34, S-100 protein, SMA, TTF1, Calretinin, and neuroendocrine markers in both regions. Graham et al. (7) performed gene detection of 4 patients with gastroblastoma and found that all of them had a MALAT1-GLI1 fusion gene, which was considered to have certain value for the diagnosis of gastroblastoma. By our literature review, a total of 8 cases have been confirmed to have the MALAT1-GLI1 fusion gene including our case. There were other fusion genes that have been found with increasing reported frequency of the disease. Such as EWSR1-CTBP1 (12), PTCH-GLI2 (13), and ACTB-GLI1 fusion gene (15).

The differential diagnosis of gastroblastoma usually includes synovial sarcoma, carcinosarcoma, gastrointestinal stromal tumor (GIST), and plexiform fibromyxoma. Gastric synovial sarcoma is rare and can show biphasic differentiation, and also have glandular and tubular structures. The synovial sarcoma is typically characterized by its highly malignant nature, displaying remarkable morphological and biological features. The specific translocation of X to chromosome 18 is an important feature of synovial sarcoma. This translocation results in fusion of the SYT gene on chromosome 18 with SSX1 (in about two thirds of cases), SSX2 (in about one third of cases), or SSX4 (in rare cases) on the X chromosome (19, 20). The incidence of carcinosarcoma is higher among the elderly population. Carcinosarcoma exhibits a malignant biological behavior, characterized by evident cellular atypia, pleomorphism, and prominent pathological mitosis. Gastrointestinal stromal tumors(GIST) exhibits a diverse range of morphological characteristics. Approximately 10% of cases show a biphasic morphology characterized by the presence of both spindle and epithelioid cells. The epithelioid cells are arranged in nests, but do not form glandular structures. The plexiform fibromyxoma is an uncommon mesenchymal tumor that primarily affects the stomach. A subset of neoplastic cells exhibit myofibroblastic differentiation, with most cases showing positive staining for SMA, MSA, and vimentin. The presence of the MALAT1-GLI1 fusion gene is identified in certain cases of plexiform fibromyxoma, exhibiting a structural resemblance to that observed in gastroblastoma (21). However, the plexiform fibromyxoma shows a benign clinical course and lacked biphasic morphology. Therefore, it is possible that MALAT1-GLI1 fusion gene in these two tumors may represent an accidental occurrence of the same genetic alteration in different tumors, rather than different subtypes of the same tumor entity.

## Conclusion

In conclusion, gastroblastoma was a low-grade malignant tumor. Postoperative pathology and immunohistochemistry were the only methods to confirm the diagnosis, and surgery is still the first choice of treatment. Among the reported cases, most of the tumors showed indolent biological behavior and the prognosis after surgery was usually good, but there were still 4 cases of local recurrence or metastasis. The patient was followed up for 24 months with no signs of recurrence or metastasis. Due to the paucity of case reports, the knowledge of this disease was still lacking. The clinical treatment effect and prognosis of gastroblastoma still needed a large number of cases and follow-up data to support.

## Data availability statement

The original contributions presented in the study are included in the article/supplementary material. Further inquiries can be directed to the corresponding authors.

## Ethics statement

Written informed consent was obtained from the individual(s) for the publication of any potentially identifiable images or data included in this article.

## Author contributions

JL: Writing – original draft. GW: Writing – review & editing. ZJ: Writing – review & editing.
